# Improved oxygenation in prone positioning of mechanically ventilated patients with COVID-19 acute respiratory distress syndrome is associated with decreased pulmonary shunt fraction: a prospective multicenter study

**DOI:** 10.1186/s40001-023-01559-9

**Published:** 2023-12-16

**Authors:** Piotr Harbut, Francesca Campoccia Jalde, Martin Dahlberg, Anders Forsgren, Elisabeth Andersson, Andreas Lundholm, Jaroslaw Janc, Patrycja Lesnik, Michal Suchanski, Pawel Zatorski, Janusz Trzebicki, Tomasz Skalec, Mattias Günther

**Affiliations:** 1 Department of Clinical Sciences Danderyd, Karolinska Institutet, Stockholm, Sweden; 2 Department of Molecular Medicine and Surgery, Karolinska Institutet, Stockholm, Sweden; 3 Department of Perioperative Medicine and Intensive Care, Thoracic Anesthesia and Intensive Care Unit, Karolinska University Hospital, Stockholm, Sweden; 4 Department of Clinical Science and Education Södersjukhuset, Karolinska Institutet, Stockholm, Sweden; 5 4th Military Clinical Hospital, Wroclaw, Poland; 6 Medical University of Warsaw, Warsaw, Poland; 7 Wroclaw Medical University, Wroclaw, Poland; 8 Department of Clinical Science and Education Södersjukhuset, Section for Anesthesiology and Intensive Care, Karolinska Institutet, Sjukhusbacken 10, SE-118 83 Stockholm, Sweden

**Keywords:** Prone position, COVID-19, Intensive care, Acute respiratory distress syndrome, Pulmonary shunt fraction

## Abstract

**Background:**

Prone position is used in acute respiratory distress syndrome and in coronavirus disease 2019 (Covid-19) acute respiratory distress syndrome (ARDS). However, physiological mechanisms remain unclear. The aim of this study was to determine whether improved oxygenation was related to pulmonary shunt fraction (Q’s/Q’t), alveolar dead space (Vd/Vtalv) and ventilation/perfusion mismatch (V’_A_/Q’).

**Methods:**

This was an international, prospective, observational, multicenter, cohort study, including six intensive care units in Sweden and Poland and 71 mechanically ventilated adult patients.

**Results:**

Prone position increased PaO_2_:FiO_2_ after 30 min, by 78% (83–148 mm Hg). The effect persisted 120 min after return to supine (*p* < 0.001). The oxygenation index decreased 30 min after prone positioning by 43% (21–12 units). Q’s/Q’t decreased already after 30 min in the prone position by 17% (0.41–0.34). The effect persisted 120 min after return to supine (*p* < 0.005). Q’s/Q’t and PaO_2_:FiO_2_ were correlated both in prone (Beta -137) (*p* < 0.001) and in the supine position (Beta -270) (*p* < 0.001). V’_A_/Q’ was unaffected and did not correlate to PaO_2_:FiO_2_ (*p* = 0.8). Vd/Vtalv increased at 120 min by 11% (0.55–0.61) (*p* < 0.05) and did not correlate to PaO_2_:FiO_2_ (*p* = 0.3). The ventilatory ratio increased after 30 min in the prone position by 58% (1.9–3.0) (*p* < 0.001). PaO_2_:FiO_2_ at baseline predicted PaO_2_:FiO_2_ at 30 min after proning (Beta 1.3) (*p* < 0.001).

**Conclusions:**

Improved oxygenation by prone positioning in COVID-19 ARDS patients was primarily associated with a decrease in pulmonary shunt fraction. Dead space remained high and the global V’_A_/Q’ measure could not explain the differences in gas exchange.

**Supplementary Information:**

The online version contains supplementary material available at 10.1186/s40001-023-01559-9.

## Introduction

About 20% of hospitalized patients [1, 2] and up to 70% of critically ill patients [2] with coronavirus disease 2019 (COVID-19) develop acute respiratory distress syndrome (ARDS), increasing mortality [3]. The majority of patients with COVID-19 ARDS require intubation and mechanical ventilation [1–3]. Prone positioning is considered as one of the most effective treatment strategies for patients with severe ARDS, it may improve oxygenation due to perfusion redistribution and more homogeneous ventilation [4, 5]. Prone positioning is used in up to 76% of mechanically ventilated COVID-19 patients and improves oxygenation in ~ 80%. This response is associated with improved survival independently of the oxygenation response [6–8]. However, the underlying physiological mechanisms of improved oxygenation remain unclear [9]. We aimed to investigate the lung physiology of prone positioning in mechanically ventilated patients with COVID-19 ARDS, and to determine whether improved oxygenation was related to three clinically accessible measures of lung function: 1. ventilation/perfusion mismatch (V’_A_/Q’), that represents the heterogeneity of ventilation and perfusion distribution in the lungs and is defined as the rate of alveolar ventilation to the rate of pulmonary blood flow. 2. The pulmonary shunt fraction (Q’s/Q’t) that is a way to determine how much perfused but poorly or not ventilated lung regions contribute to hypoxemia in arterial blood. 3. The alveolar dead space (Vd/Vtalv) that represents the volume of air present in the respiratory zone of the lungs not taking part in gas exchange. In lung disease, alveolar dead space corresponds to alveoli that are ventilated, but not perfused by the pulmonary circulation. In a brief report, hypoxemia in COVID-19 ARDS correlated to dead space but was dissociated from lung mechanics [10]. A report of 10 intubated Covid-19 patients monitored with Electrical Impedance Tomography suggested that the observed elevated ventilation-perfusion mismatch was more due to lung units with dead space than shunt [11]. A report of 12 patients suggested that prone positioning had little effect on dead space fraction [12]. In previous studies, we reported that proning increased PaO_2_:FiO_2_ mainly in patients with PaO_2_:FiO_2_ < 120 mmHg [13] and Q’s/Q’t and Vd/Vtalv increased in early mild to moderate COVID-19, but their relative contributions were highly variable (14). We hypothesized that prone positioning would reduce V’_A_/Q’, Q’s/Q’t and Vd/Vtalv.

## Materials and methods

All procedures performed in studies involving human participants were in accordance with the ethical standards of the institutional and national research committees and with the 1964 Helsinki Declaration and its later amendments. The study was approved by the Swedish Ethical Review Authority (2020-02593), the Bioethical Commission, Military Medical Chamber, Poland (KB7/20/178/29), the Bioethical Council, Medical University of Warsaw, Poland (AKBE/219/2020) and the Bioethical Commission, Wrocław Medical University, Poland (KB-764/2020). Patient informed consent was granted as per the approvals. All data were de-identified following collection.

From three centers in Sweden (Södersjukhuset, Danderyd Hospital, Karolinska University Hospital) and three centers in Poland (4-th Military Clinical Hospital in Wroclaw, Medical University of Warsaw, Wroclaw Medical University), we included 71 consecutive adult (> 18 yr) ARDS patients who, while mechanically ventilated, received at least one session of prone positioning, lasting more than or equal to 12 h. All patients had documented COVID-19-positive reverse transcriptase polymerase chain reaction tests from either upper airway swab or bronchoalveolar lavage. All patients fulfilled the Berlin definition of ARDS [15]. The decision to initiate proning and the timing were based on the American–European Consensus Conference criteria for severe ARDS (PaO_2_:FiO_2_ ratio of < 150 mm Hg, with a FiO_2_ of ≥ 0.6) [16] and our experience with COVID-19 ARDS [13]. PaO_2_:FiO_2_ ratio was used in the present study to assess changes of pulmonary gas exchange in response to the prone position. This ratio is commonly used in ARDS severity assessment and as a marker of disease progression or to compare patients with different FiO2 (Feiner et al, PMID 27618274 [17]). Patients were included between July 2020 and April 2021. Data were collected before the proning session (baseline), at 30 and 120 min after initiation of proning, just before returning the patient to supine, and 30 and 120 min after returning to supine position. The high temporal resolution of parameters was intended to enable investigation of the pulmonary effects related to the proning intervention. We hypothesized that the response to the first proning session would be the most important indicator of the occurring physiological changes and allow for comparison of the entire patient cohort, based on our earlier experience [13]. Data included respiratory mechanics, ventilatory data, gas exchange and hemodynamic parameters, in addition to demographic and anthropometric variables. Follow-up was conducted at 30 days from the proning session. We intended to include all patients who were mechanically ventilated and proned. However, due to lack of availability of the equipment to measure cardiac output in this intensive phase of the pandemic, we could not include all eligible patients. The selection was random and was not due to patient characteristics.

### Physiologic calculations

The Q’s/Q’t was calculated using the equation:

$${\text{Q}}^{\prime} {\text{s}}/{\text{Q}}^{\prime} {\text{s}}{\mkern 1mu} \, = \,{1}00{\text{x}}\left[ {\left( {{\text{O}}_{2} {\text{Ideal}} - {\text{CaO}}_{2} } \right)/\left( {{\text{O}}_{2} {\text{Ideal}} - {\text{PcvO}}_{2} } \right)} \right]$$

O_2_Ideal was calculated by:

$${\text{O}}_{2} {\text{Ideal}}\, = \,{1}.{\text{39xHb}}\, + \,0.00{\text{31x}}\,\left[ {\left( {{\text{FiO}}_{2} \, \times \,{713}} \right) - {\text{PaCO}}_{2} } \right)]$$

The fraction of alveolar dead space ventilation (Vd/Vtalv) was calculated using the modified Bohr-Enghoff equation:

$${\text{Vd}}/{\text{Vt}}\, = \,\left( {{\text{PaCO2}} - {\text{PECO}}_{2} } \right)/{\text{PaCO}}_{2}$$

Where PaCO_2_ was a partial pressure of carbon dioxide in arterial blood and PECO_2_ the tension of the expired carbon dioxide (used as a surrogate of the mixed expired carbon dioxide in the original Bohr-Enghoff formula) and calculated from the equation PECO_2_ = FECO_2_ x (P_bar_–P_H2O_). P_bar_ and P_H2O_—ambient conditions of the barometric pressure and the partial pressure of water vapor, stated at 760–47 = 713 (mmHg). The fraction of expired carbon dioxide (FECO_2_) was derived as a function of the carbon dioxide production (VCO_2_) and minute ventilation of the lungs (VE), from the equation:

$${\text{FECO}}_{2} \, = \,{\text{VCO}}_{2} /{\text{VE}}$$

The VE was calculated by subtracting the apparatus dead space and the anatomical dead space from the measured tidal volumes (milliliters), multiplied by the respiratory rate (per minute). The anatomical dead space was calculated according to the modified equation proposed by Nunn [20]:

$${\text{VE}}\, = \,\left( {{2}.{\text{2xPBWx}}0.{5}\, + \,{\text{DSPappx}}0.00{1}} \right){\text{xRR}}$$

The predicted body weight (PBW) was calculated according to the ARDS-net and the apparatus dead space (DSPapp) was study-site specific [18].

Due to the unavailability of volumetric capnometry data, VCO_2_ was computed from the oxygen consumption (VO_2_) and respiratory quotient (RQ) (arbitrarily set at 0.8). CO—measured cardiac output. CaO_2_—arterial oxygen content. CcvO_2_—central venous oxygen content used as a surrogate of the mixed venous oxygen content.

$${\text{VCO}}_{2} \, = \,{\text{VO}}_{2} {\text{xRQ}},\quad {\text{where VO}}_{2} \, = \,\left( {{\text{CaO}}_{2} - {\text{CcvO}}_{2} } \right){\text{xCO}}$$

The Vd/Vt and Q’s/Q’t were calculated individually for all patients at protocol-derived measurement points and PaO_2_ and PaCO_2_ values were corrected for temperature, according to Bradley’s correction factor [19]. We calculated ventilation/perfusion ratio V’_A_/Q’ by dividing the estimated alveolar ventilation by the cardiac output, measured with the pulse contour analysis (FloTrac system, Edwards). The Ventilatory ratio was calculated using the equation [20]:

$${\text{VR}}\, = \,\left[ {{\text{minute ventilation }}\left( {{\text{ml}}/{\text{min}}} \right)\, \times \,{\text{Pa}}_{{\text{CO2}}} \left( {{\text{mmHg}}} \right)} \right]/\left( {{\text{predicted body weight}}\, \times \,{1}00\, \times \,{37}.{5}} \right)$$

The Oxygenation index was calculated using the Eq. (20):

$${\text{OI}}\, = \,{\text{mean airway pressure MAP}}\, \times \,{\text{FiO}}_{2} \, \times \,{1}00/{\text{PaO}}_{2}$$

### Statistical analyses

Statistical analyses were done using GraphPad Prism version 9.4.0 (GraphPad Software) and R (v 3.5.1). The primary outcome was the Q’s/Q’t. All continuous data are presented as medians with interquartile range (IQR). All temporal data sets were analyzed with mixed effects model (REML), fixed effects (type III) and Tukey's multiple comparisons test. A GEE model with an autoregressive structure (AR-1) was used for correlation analyses under an assumption of linear function of regressors and gaussian residuals. We inspected residual plots to exclude large deviations from normality, under the assumption that gaussian models are usually stable under minor such deviations. Regression models used to predict PaO_2_:FiO_2_ included available baseline variables thought to have possible causal effects of respiration and circulation. *α* = 0.05 was considered significant. *p < 0.05, **p < 0.01, ***p < 0.005, ****p < 0.001.

## Results

The characteristics of the participants at study inclusion are presented in Table 1. All diseases reported were already present at the time of admission. The observed mortality of 63% was higher than the one estimated by the average SAPS III of 64 points, which would predict a mortality rate of 43%.

**Table 1:** Subject characteristics on inclusion

Characteristic	*N* = 71^1^
^a^Female	14/71 (20%)
^b^Age (years)	66.0 (55.0, 73.0)
^b^Weight (kg)	83.0 (75.5, 91.0)
^b^Predicted body weight (kg)	70.6 (64.2, 75.1)
^b^Height (cm)	175.0 (168.5, 180.0)
^a^Death	45/71 (63%)
^b^SAPS III	64.0 (60.0, 73.5)
^a^Diabetes mellitus II	16/71 (23%)
^a^Hypertension	34/71 (48%)
^a^Hyperlipidemia	6/71 (8.5%)
^a^Asthma	5/71 (7.0%)
^a^COPD	6/71 (8.5%)
^a^OSAS	3/71 (4.2%)
^a^Obesity	7/71 (9.9%)
^a^Cardiovascular	12/71 (17%)
^a^Cancer	7/71 (9.9%)
^a^Kidney failure	1/71 (1.4%)
^a^Transplant	6/71 (8.5%)

^a^*n*/*N* (%)

^b^Median (IQR)

The ventilatory, metabolic and circulatory data from the first prone positioning session are presented in Table 2.

**Table 2:** Ventilatory, metabolic and circulatory data for the first proning session

Median (IQR)	Baseline supine	Prone at 30 min	Prone at 120 min	Prone before turn	Supine at 30 min	Supine at 120 min	*p*
Ventilatory parameters							
PaO_2_:FiO_2_ (mmHg)	83 (68–111)	113 (91–160)	114 (94–163)	148 (114–188)	106 (92–145)	129 (100–150)	< 0.001
FiO_2_	0.85 (0.75–1)	0.8 (0.6–0.9)	0.7 (0.55–0.85)	0.6 (0.45–0.7)	0.65 (0.6–0.8)	0.6 (0.5–0.75)	< 0.001
PaO_2_ (mmHg)	67 (59–78)	80 (69–97)	79 (67–91)	76 (68–90)	72 (63–79)	76 (63–84)	0.002
PaCO_2_ (mmHg)	51 (45–56)	53 (45–64)	51 (45–60)	50 (44–57)	51 (46–59)	53 (43–58)	0.14
Tidal volume (mL)	500 (430–572)	530 (440–620)	560 (450–660)	540 (470–650)	530 (450–600)	530 (470–610)	0.06
Tidal volume (mL/kg PBW)	7.1 (6.2–8.7)	7.8 (6.4–9.2)	8.2 (6.7–9.7)	8.2 (6.9–9.2)	7.7 (7–8.4)	7.8 (6.8–9)	0.08
Respiratory rate (bpm)	21 (15–23)	22 (15–24)	20 (15–23)	20 (16–24)	20 (17–23)	19 (16–23)	0.14
Minute ventilation (L/min)	10 (7.8–11.5)	10.8 (8.3–12.3)	11 (9.5–12.2)	11.1 (9–13)	10.7 (9–12.1)	11 (8.7–12)	0.01
Alveolar ventilation (L/min)	6.8 (5.8–8)	7.3 (5.9–8.9)	7.7 (6.6–9.2)	7.8 (6.5–9.5)	8 (6.3–9.1)	7.9 (6.5–9)	0.01
PEEP (cmH_2_O)	12 (10–14)	12 (10–14)	12 (10–13)	12 (10–13)	12 (10–14)	12 (10–14)	0.90
Plateau pressure (cmH_2_O)	24 (22–28)	26 (23–27)	26 (24–27)	25 (23–28)	25 (23–28)	26 (23–29)	0.59
Cdyn (mL/cmH_2_O)	38 (30–48)	36 (31–54)	39 (32–54)	40 (35–47)	41 (33–51)	39 (31–51)	0.77
Oxygenation index (units)	21 (15–28)	13 (11–20)	13 (9–19)	12 (9–16)	17 (12–20)	14 (10–19)	0.001
Ventilatory ratio (units)	1.9 (1.6–2.4)	2.1 (1.7–2.8)	2.1 (1.7–2.7)	3.0 (2.5–4.1)	2.2 (1.8–2.5)	2.2 (1.8–2.5)	< 0.001
Neuromuscular blockers (*n*)	25	18	18	16	15	14	0.14
Metabolic parameters							
Arterial pH	7.37 (7.32–7.42)	7.35 (7.29–7.40)	7.36 (7.30–7.40)	7.39 (7.33–7.45)	7.38 (7.35–7.44)	7.40 (7.34–7.45)	0.08
Base excess	2.9 (0–6)	3 (−0.9 to 6)	3 (−0.5 to 6.8)	4.3 (1.7–9.5)	5.3 (1.8–9.5)	5.8 (2.9–10.8)	0.006
Circulatory parameters							
Cardiac output (L/min)	6.1 (4.7–7.5)	6.6 (4.7–7.7)	5.9 (4.7–7.4)	6.3 (5.4–7.4)	6.4 (5.3–7.5)	6.3 (4.9–7.8)	0.56
Mean arterial pressure (mmHg)	80 (70–89)	82 (75–88)	81 (74–90)	83 (72–95)	82 (74–89)	81 (71–95)	0.50
Norepinephrine (µg/kg/min)	0.06 (0.02–0.14)	0.07 (0.03–0.14)	0.06 (0.02–0.12)	0.04 (0.01–0.1)	0.06 (0.01–0.11)	0.05 (0.01–0.12)	0.19
Time in prone position (h)		19 (14.5–21)					

**Fig. 1:**
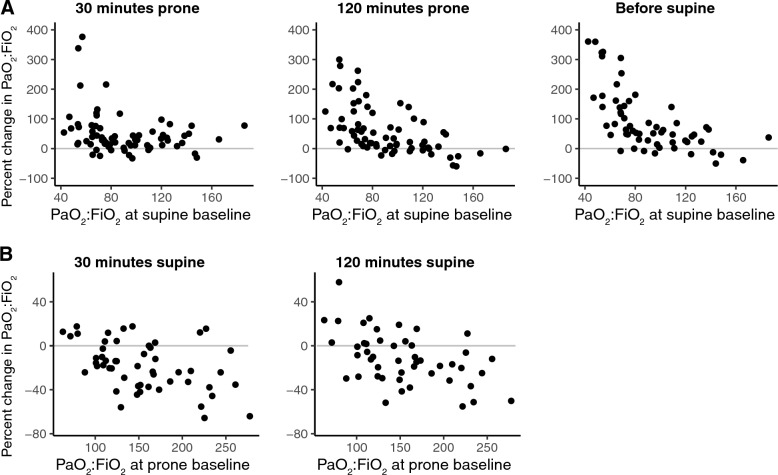
Individual patient values for **A**: percent change of PaO_2_:FiO_2_ in relation to baseline. **B**: percent change of PaO_2_:FiO_2_ in relation to prone baseline (prone position just before return)

The percent changes in PaO_2_:FiO_2_ were plotted as a function of baseline PaO_2_:FiO_2_, at 30 min, 120 min, and at the end of the proning period (just before returning to supine), as well as the percent changes in PaO_2_:FiO_2_ from the end of proning (prone baseline) to 30 min and 120 min in supine position. Improvements from baseline PaO_2_:FiO_2_ were larger for low baseline PaO_2_:FiO_2_, and more pronounced with time. When returning from prone to supine position, patients with a relatively higher PaO_2_:FiO_2_ at prone baseline decreased as much as 100 units back to supine. Patients with lower improvement of PaO_2_:FiO_2_ did not drop as much in PaO_2_:FiO_2_ when returning to supine (Fig. 1A, B).

**Fig. 2:**
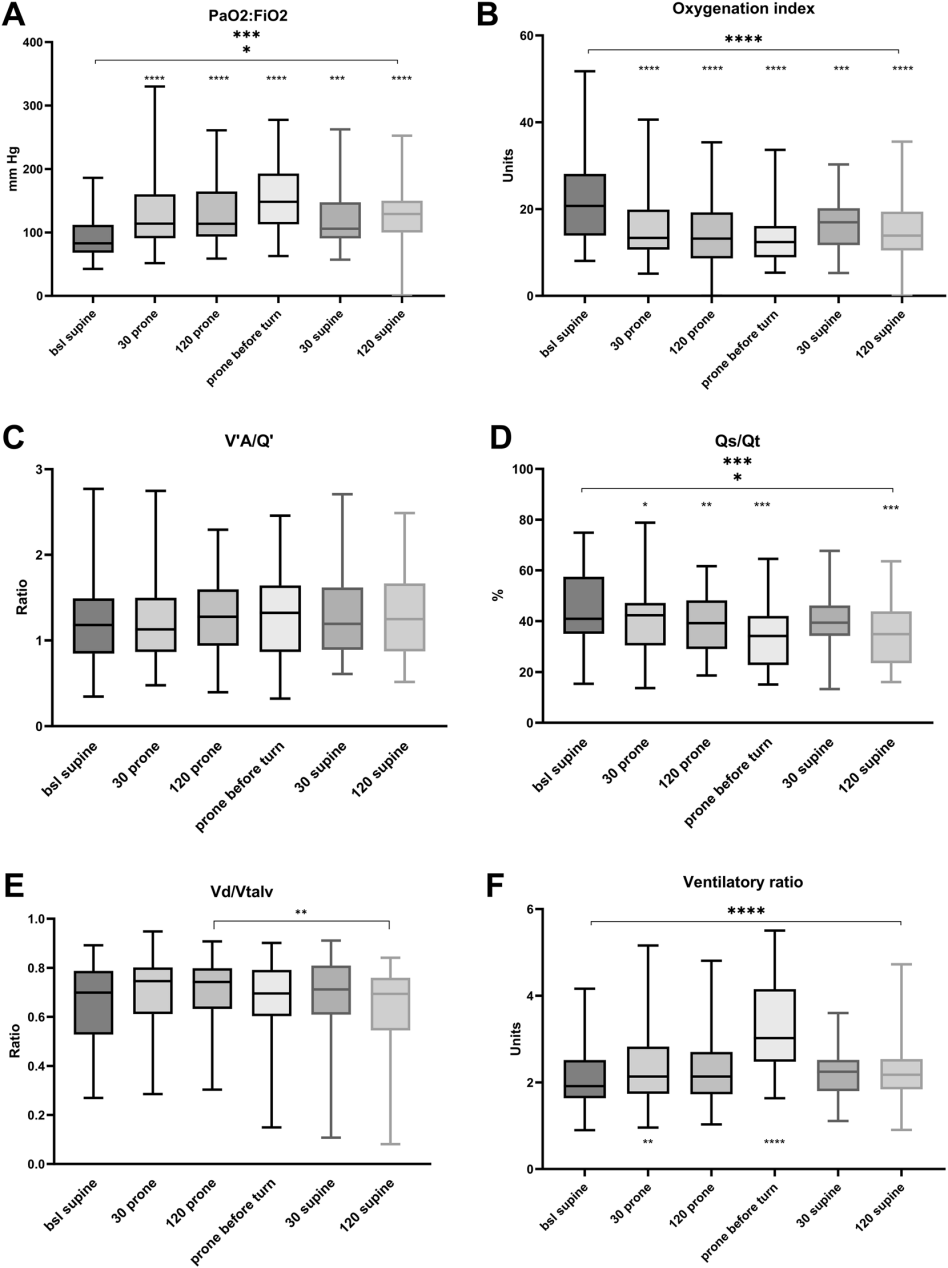
Pulmonary physiology for six time points during proning. **A**: PaO_2_:FiO_2_. B: oxygenation index. **C** V’_A_/Q’ (ventilation/perfusion ratio). **D**: Q’s/Q’t (pulmonary shunt fraction). **E** Vd/Vtalv (ratio of airway dead space to alveolar tidal volume). **F** ventilatory ratio. Displayed as medians with IQR. Asterisks mark in comparison to baseline, if not specifically marked with lines. *p < 0.05, **p < 0.01, ***p < 0.005, ****p < 0.001

The pulmonary physiological response to proning is shown in Fig. 2. Proning increased PaO_2_:FiO_2_, starting at 30 min from initiation, by a maximum of 78% (83–148 mm Hg). The effect remained throughout the period of study observation, up to 120 min after return to supine (*p* < 0.001) (Fig. 2A). Oxygenation index decreased at 30 min after prone positioning by a maximum of 43% (21–12 units) and remained decreased throughout the observation period (Fig. 2B). V’A/Q’ remained unchanged (Fig. 2C). Q’s/Q’t decreased during proning by a maximum of 17% (0.41–0.34). The effect was consistent to the end of the observation and was still improved 120 min after return to supine (*p* < 0.005) (Fig. 2D). Vd/Vtalv remained unchanged in comparison to baseline and differed at 120 min after initiation of proning in comparison to 120 min after returning to supine by 13% (0.71–0.63) (*p* < 0.01) (Fig. 2E). Ventilatory ratio increased at 30 min after initiation of proning (*p* < 0.01) and continued to increase before returning to supine (*p* < 0.001) to a maximum of 58% of baseline (1.9–3.0) (Fig. 2F). Individual responses are shown in Additional file 1: Figure S1.

**Fig. 3:**
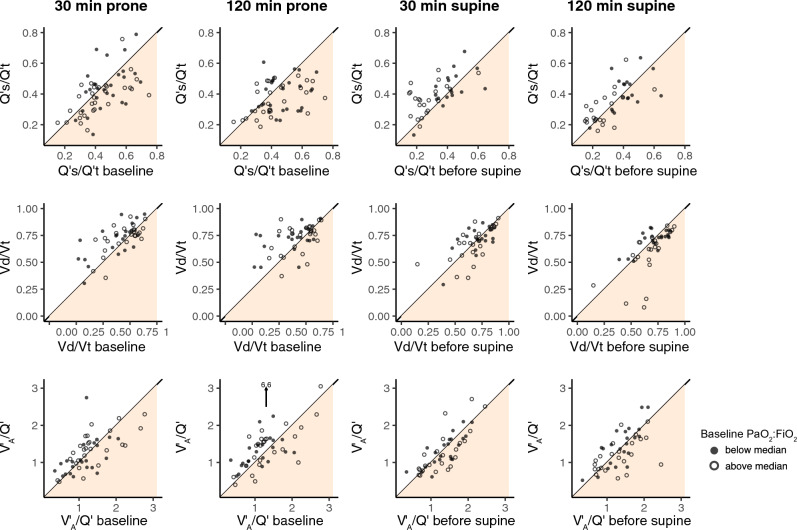
The relationship between Q’s/Q’t, Vd/Vtalv, V’_A_/Q’, and their supine and prone baselines (prone position just before return). Points close to the diagonal indicate no change. Values below the diagonal indicate decrease, and values above the diagonal indicate increase. Baseline PaO_2_:FiO_2_ below median marked as dots, PaO_2_:FiO_2_ above median marked as circles. *P*-values which were not significant are specified in exact numbers

Changes in Q’s/Q’t, Vd/Vtalv, and V’_A_/Q’ were visualized in scatter plots, showing individual patient values. The higher the distance from the diagonal, the higher the decrease in Q’s/Q’t on proning (white area) or increase in Q’s/Q’t (beige area), while returning to supine position (Fig. 3). The highest level of scatter (variance) was detected in Q’s/Q’t.

The correlation of time dependent variables with PaO_2_:FiO_2_ was then investigated for the intervention supine to prone position. In prone position, a strong negative correlation between Q’s/Q’t and PaO2:FiO2 was detected, with an increase in PaO_2_:FiO_2_ of 137 units per unit Q’s/Q’t decrease in the linear approximation (Beta = 137, *p* < 0.001). Vd/Vtalv and V’_A_/Q’ did not correlate to PaO_2_:FiO_2_ (*p* = 0.3 and *p* = 0.8, respectively). When returning from prone to supine position, an even stronger correlation between Q’s/Q’t and PaO_2_:FiO_2_ was detected (Beta -270, *p* < 0.001), meaning that for every increase in Q’s/Q’t, PaO_2_:FiO_2_ decreased 270 units. Neither Vd/Vtalv (*p* = 0.6) nor V’A/Q’ (*p* = 0.8) did correlate to PaO_2_:FiO_2_. We identified PaO_2_:FiO_2_ at baseline as the only variable predictive of the PaO_2_:FiO_2_ at the different time points after proning (Beta 1.3; *p* < 0.001 and the other tested variables in Table 3).

**Table 3:** Pulmonary physiological variables in proning response (PaO_2_:FiO_2_), using time dependent variables correlation, showing a high correlation between Q’s/Q’t and PaO_2_:FiO_2_ in both proning and return to supine

Characteristic	Beta	95% CI^a^	*p*-value
From supine to prone (PaO_2_:FiO_2_)			
Q’s/Q’t	−137	−275	< 0.001
Vd/Vtalv	25	−21, 72	0.3
V’_A_/Q’	1.4	-7.5, 10	0.8
From prone to supine (PaO_2_:FiO_2_)			
Q’s/Q’t	−270	−540	< 0.001
Vd/Vtalv	−22	−108, 64	0.6
V’_A_/Q’	−2.2	−25, 21	0.8
Predictor of PaO_2_:FiO_2_ at 30 min prone			
PaO_2_:FiO_2_ at baseline	1.3	0.83, 1.7	< 0.001
Vd/Vtalv at baseline	−27	−113, 60	0.55
Q’s/Q’t at baseline	69	−28, 166	0.17
Male	−25	−60, 10	0.17
Cardiovascular/HT	−2.8	−29, 23	0.83
COPD/asthma/OSAS	−4.2	−39, 31	0.81
Predictor of PaO_2_:FiO_2_ at 120 min prone			
PaO_2_:FiO_2_ at baseline	0.04	−0.45, 0.52	0.88
Vd/Vtalv at baseline	−16	−117, 84	0.75
Q’s/Q’t at baseline	−34	−147, 78	0.55
Male	−0.86	−42, 40	0.97
Cardiovascular/HT	−8.9	−39, 22	0.57
COPD/asthma/OSAS	−6.6	−47, 34	0.75
Predictor of PaO_2_:FiO_2_ before turn to supine			
PaO_2_:FiO_2_ at baseline	−0.18	−0.69, 0.33	0.49
Vd/Vtalv at baseline	−57	−162, 47	0.29
Q’s/Q’t at baseline	−50	−172, 71	0.42
Male	16	−27, 58	0.48
Cardiovascular/HT	19	−15, 52	0.28
COPD/asthma/OSAS	11	−31, 53	0.61

V’_A_/Q’ and Vd/Vtalv did not correlate to PaO_2_:FiO_2_. Predictors of PaO_2_:FiO_2_ at 30, 60 min after proning, and before turn to supine. For every increase in PaO_2_:FiO_2_ at baseline, PaO_2_:FiO_2_ increased 1.3 units at 30 min after proning. Other characteristics did not correlate to changes in PaO_2_:FiO_2_

^a^*CI* Confidence Interval

## Discussion

In this study, we show that the oxygenation improvement by prone positioning was primarily associated with a decrease in pulmonary shunt fraction, in mechanically ventilated patients with COVID-19 associated ARDS.

COVID-19 ARDS is a heterogeneous disease. The lung morphology is characterized by coexisting signs of alveolar damage and interstitial injury: ground-glass opacity with or without consolidation and septal thickening are common findings on CT images [22]. Progression of pulmonary injury is characterized by alterations of the pulmonary vasculature tree, with dynamic increase in the size of vessels [23, 24]. Both alveolar and vascular pathology exist in early, less severe COVID-19 and the late, fatal cases [14, 25]. It is possible that the heterogenous nature of the disease causes diverse physiological responses in the lung, before and after the established method of prone positioning.

We investigated the physiological effects of proning as treatment intervention initiated by the caregiver, in a cohort of mechanically ventilated COVID-19 patients with moderate to severe ARDS, according to the Berlin definition of ARDS [15]. Median PaO_2_:FiO_2_ at time of intervention was 83 mmHg, a lower starting point than in our previous report [13]. This may reflect that the decision to prone was taken later, or that the cohort was more severely ill. Also, the time in prone position was longer than in our previous report, 19 h, compared to 14.5 h [13].

First, we evaluated the change in PaO_2_:FiO_2_ as a function of baseline PaO_2_:FiO_2_. Changes were both higher and more pronounced over time for patients with low baseline PaO_2_:FiO_2_. Proning improved PaO_2_:FiO_2_, starting at 30 min, and the improvement was consistent throughout the observation period. The 82% (47–148 mm Hg) increase (Additional file 1: Table S1) was higher than the 55–60% previously reported [8, 13]. Hence, we detected a heterogeneity in response and the patients with a more severe ARDS responded better to prone positioning, in line with previous investigations [8]. When returning to supine, patients who had a higher improvement in oxygenation in prone position lost up to 100 mmHg in PaO_2_:FiO_2_. Most patients with a lower improvement in PaO_2_:FiO_2_ did not lose as much oxygenation when turned back to supine. Thus, the reversibility of the response to proning was higher in patients with a more severe ARDS. Proning also caused a decrease in oxygenation index, which describes the severity of hypoxic respiratory failure including the mean airway pressure, an important determinant of oxygenation [21].

Next, we evaluated changes in pulmonary physiology during and after proning. Primarily, we investigated three established variables of pulmonary function: Q’s/Q’t, V’_A_/Q’ and Vd/Vtalv [26]. The rationale for selecting these parameters was that they could be obtained in clinical practice and did not require invasive procedures or transfers, which could jeopardize patient safety, especially in a cohort with high mortality (63% in this cohort). We primarily calculated Q’s/Q’t, which decreased by 7 percentage points (41% before proning to 34% before returning to supine). Interestingly, the effect lasted throughout the observation period, also when returning to baseline. Q’s/Q’t is quantified as fraction of cardiac output distributed to nonventilated units. ARDS of other origins is characterized by severe hypoxemia due to shunt that might exceed 50% [27]. Q’s/Q’t and PaO_2_:FiO_2_ were highly correlated for the intervention supine to prone position, and for every unit decrease in Q’s/Q’t, the PaO_2_:FiO_2_ increased 137 units (*p* < 0.001), which further confirmed the pathophysiological mechanism behind the improvement in oxygenation.

V’_A_/Q’ varied between 1.13 and 1.32, it did not change significantly in prone position, and it did not correlate to PaO_2_:FiO_2_ for the intervention supine to prone position or when returning to supine. The Riley three-compartment model is convenient [28], but assumes that the effects of V’_A_/Q’ mismatch on PaO_2_ and PaCO_2_ are entirely due to shunt and physiological dead space ventilation [26] and ignores gas exchange in units with other V’_A_/Q’ ratios, which may be assessed with multiple inert gas elimination technique [29]. Also, the calculated shunt corresponds to the amount of shunt of mixed venous blood that would result in the observed arterial oxygenation in the absence of low V’_A_/Q’ regions. But venous admixture may be increased even in the absence of true shunt [26]. In our study cohort, alveolar ventilation and cardiac output remained relatively unchanged. Thus, the global V’_A_/Q’ measure was not sensitive enough to explain the differences in gas exchange.

Vd/Vtalv varied between 0.69 and 0.75 and did not correlate to PaO_2_:FiO_2_ for the intervention supine to prone position, thus confirming previous findings suggesting that prone positioning had little effect on dead space fraction, although dead space decreased at 120 min after returning to supine, in comparison to 120 min after initiation of proning [12]. Despite its well-established role in lung physiology, dead space is not routinely assessed in critically ill patients [30]. In COVID-19 ARDS, increased alveolar dead space may be related to obstruction of small pulmonary arteries due to microthrombosis [25], and is associated with high D-dimer levels and a lower likelihood of being discharged alive [31]. Different methodological approaches have been used for the calculation of the physiological dead space in ICU settings, where reliable VCO_2_ measurements are challenging to obtain [32, 33]. The Ventilatory ratio is a useful estimate of impaired lung ventilation in terms of CO_2_ elimination, it is strongly correlated to dead space in ARDS, and it is associated with increased risk of an adverse outcome [20]. The Ventilatory ratio increased 1.1 units during prone positioning (from 1.9 at baseline to 3 before return to supine). Earlier studies reported a lower increase (of 0.03 units) on proning [8].

Q’s/Q’t, Vd/Vtalv, and V’_A_/Q’ were then visualized in scatter plots, showing changes in the individual patients. The highest level of variance was detected in Q’s/Q’t, confirming that Q’s/Q’t was the physiologic parameters mostly affected by proning. Possible predictors of the oxygenation response were then evaluated. PaO_2_:FiO_2_ at baseline predicted PaO_2_:FiO_2_ at 30 min and no further variables proved to correlate to changes in PaO_2_:FiO_2_, confirming that baseline oxygenation may predict patient response.

The assumed physiological mechanisms of the improved oxygenation may be explained in part by previously published experimental studies. Using positron emission tomography imaging of nitrogen in sheep, prone position was shown to improve gas exchange by restoring aeration while preserving perfusion in dorsal lung regions, and by making the distribution of ventilation more uniform [34]. While we could not assess the specific contribution of different mechanisms, we detected a decrease in shunt, and it is possible that proning also improved the distribution of ventilation, although the global V’_A_/Q’ index was not sufficiently sensitive to detect it. Prone positioning was previously shown to increase transpulmonary pressures while improving oxygenation and hemodynamics in patients with moderate to severe ARDS [35], which may be a mechanism of improvement also in COVID-19 ARDS.

There are some limitations to be discussed. First, there was no prespecified sample size for the cohort enrolled since, at the study time, no knowledge was available regarding gas exchange response or physiological variable changes to be expected in Covid-19 ARDS patients during prone positioning. Second, a potential inclusion bias may exist since the initiation of prone positioning may ultimately reflect the practice of the treating clinical team. Differences in patient selection among centers may potentially reflect different resource availability at the time of the pandemic. The issues of staffing, burden of patients, and ICU occupancy may have affected the timeliness of delivery of proning and the use of the non-invasive ventilatory support prior to intubation. Third, this study was not powered to determine survival, so the reported mortality data are not a predefined outcome variable of interest in our study. Fourth, the assumptions and populational data-based gas exchange modelling are clinically applicable but may require further investigations of validity. However, it should be noted that the investigation was performed during a severe phase of the pandemic, and the patients in need of prone positioning were severely ill. Therefore, further investigations which would require a lung physiology laboratory were not possible, and we believe that the results reflect important insights of changes occurring in the lungs as a result of proning. Fifth, although PaO_2_:FiO_2_ is a surrogate of venous admixture and thus correlates with improvements in pulmonary shunt fraction, differences in FiO_2_ and cardiac output may substantially influence venous admixture and thus the calculated pulmonary shunt fraction [36]. At a given venous admixture, the PaO_2_:FiO_2_ ratio may differ, depending on oxygen consumption and cardiac output. Conversely, for the same PaO_2_:FiO_2_, venous admixture may vary with FiO2, while cardiac output did not differ depending on proning in our cohort (*p* = 0.56). Selecting PEEP according to PaO_2_:FiO_2_ ratio may also be misleading if hemodynamics are not taken into account. PEEP was not changed throughout the study neither in relation to PaO_2_:FiO_2_ nor to prone positioning (*p* = 0.90). Sixth, tidal volumes were higher (mean 7.1–8.2) than the recommended < 6 mL/kg PBW for lung protective ventilation [37]. While a confounder of the PaO_2_:FiO_2_ by increased tidal volumes cannot be excluded, tidal volumes were not affected by proning (*p* = 0.8). Seventh, the choice of the ventilator mode and use of neuromuscular relaxants were decisions of the attending clinician. Therefore, the ventilation strategy differed among patients but was largely kept unchanged within patients throughout the study period (Additional file 1: Table S3). For this reason, no conclusion can be drawn regarding the effect of mode of ventilation or neuromuscular blockers on the study results.

## Conclusions

Improved oxygenation by prone positioning in COVID-19 ARDS patients was primarily associated with a decrease in pulmonary shunt fraction. Dead space remained high and the global V’_A_/Q’ measure could not explain the differences in gas exchange.

### Supplementary Information

**Additional file 1: Figure S1. **Individual temporal responses to proning. A. PFI, B. VdVt, C. Qs/Qt, D. VA/Q. Blue area is standard deviation. **Table S1.** Pulmonary physiology values for six timepoints during the first proning session. Median (IQR). Qs/Qt (pulmonary shunt). B. Vd/Vt (ratio of airway dead space to alveolar tidal volume). C. VA/Q (Ventilation-perfusion ratio), D. PFI. **Table S2.** Determination of responders to prone positioning based on PaO2:FiO2 increase greater than or equal to 20mmHg, dynamic compliance (Cdyn) improvement, Ventilatory Ratio and CO2 decrease. Δ% = relative percent change. @30min= timepoint 30minutes after proning. @16h = timepoint 16 hours after proning. **Table S3. **Ventilation modes used during the study.

## Data Availability

The datasets used and/or analyzed during the current study are available from the corresponding author on reasonable request.
