# Supplementary material for: Improved oxygenation in prone positioning of mechanically ventilated patients with COVID-19 acute respiratory distress syndrome is associated with decreased pulmonary shunt fraction: a prospective multicenter study
Source: Eur J Med Res. 2023 Dec 16;28:597. doi: 10.1186/s40001-023-01559-9 (PMC10725003; doi:10.1186/s40001-023-01559-9)
Supplement: Supplementary file 1 — Additional file 1: Figure S1. Individual temporal responses to proning. A. PFI, B. VdVt, C. Qs/Qt, D. VA/Q. Blue area is standard deviation. Table S1. Pulmonary physiology values for six timepoints during the first proning session. Median (IQR). Qs/Qt (pulmonary shunt). B. Vd/Vt (ratio of airway dead space to alveolar tidal volume). C. VA/Q (Ventilation-perfusion ratio), D. PFI. Table S2. Determination of responders to prone positioning based on PaO2:FiO2 increase greater than or equal to 20mmHg, dynamic compliance (Cdyn) improvement, Ventilatory Ratio and CO2 decrease. Δ% = relative percent change. @30min= timepoint 30minutes after proning. @16h = timepoint 16 hours after proning. Table S3. Ventilation modes used during the study. [file 40001_2023_1559_MOESM1_ESM.pdf]

Supplemental figure 1

Individual temporal responses to proning. A. PFI, B. VdVt, C. Qs/Qt, D. V_A/Q. Blue area is standard deviation.

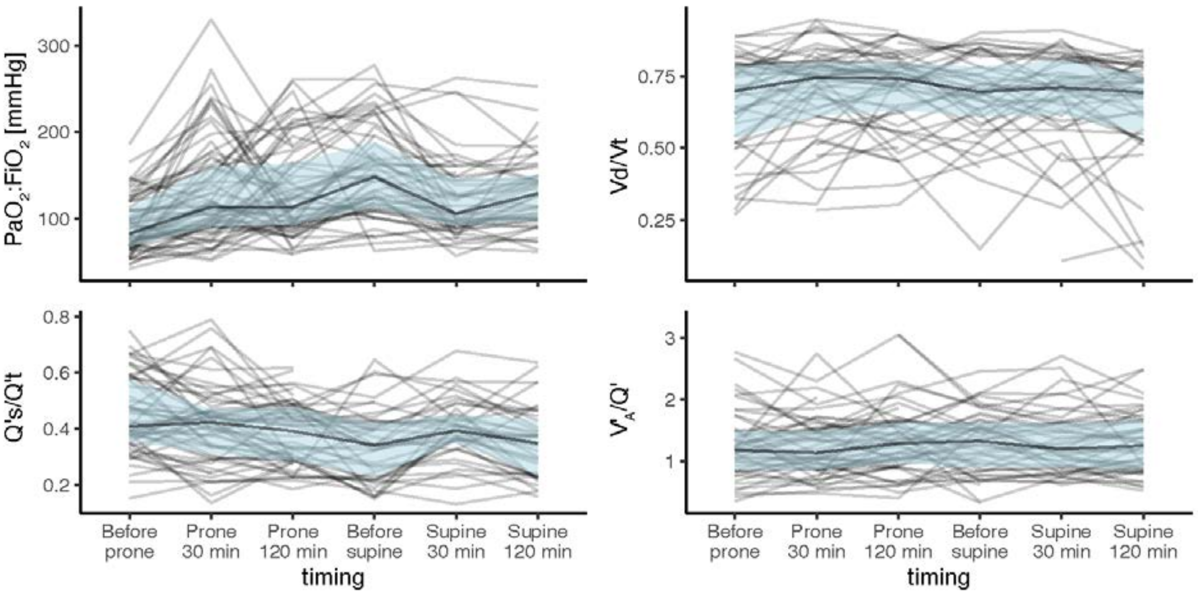

Supplemental Table 1

Pulmonary physiology values for six timepoints during the first proning session. Median (IQR). Qs/Qt (pulmonary shunt). B. Vd/Vt (ratio of airway dead space to alveolar tidal volume). C. V_A/Q (Ventilation-perfusion ratio), D. PFI.

Characteristic[1]	bsl supine, N = 71	30 prone, N = 71	120 prone, N = 71	prone before turn, N = 71	30 supine, N = 71	120 supine, N = 71
Q’s/Q’t	0.41 (0.36, 0.57)	0.42 (0.31, 0.47)	0.39 (0.29, 0.48)	0.34 (0.23, 0.42)	0.39 (0.35, 0.45)	0.35 (0.24, 0.43)
Unknown	12	13	15	26	30	30
Vd/Vtalv	0.55 (0.46, 0.65)	0.56 (0.47, 0.67)	0.61 (0.49, 0.69)	0.59 (0.47, 0.69)	0.62 (0.50, 0.67)	0.61 (0.54, 0.65)
Unknown	14	7	12	20	25	26
V'_A/Q'	1.18 (0.87, 1.49)	1.13 (0.87, 1.48)	1.28 (0.96, 1.58)	1.32 (0.89, 1.61)	1.19 (0.90, 1.60)	1.25 (0.87, 1.66)
Unknown	21	14	16	23	24	24
$PaO_2:FiO_2$	83 (68, 111)	114 (91, 160)	114 (94, 164)	148 (114, 188)	106 (92, 145)	129 (100, 150)
Unknown	2	2	1	11	19	23

[1]Median (IQR)

Supplemental table 2

Determination of responders to prone positioning based on PaO_2:FiO_2 increase greater than or equal to 20mmHg, dynamic compliance (Cdyn) improvement, Ventilatory Ratio and CO_2 decrease.
Δ% = relative percent change. @30min= timepoint 30minutes after proning. @16h = timepoint 16 hours after proning.

Prone response	PaO_2/FiO_2	Cdyn	Ventilatory Ratio	CO2
Responders @30min; % (N/tot)	61 (42/69)	46 (27/59)	33 (22/67)	28 (19/68)
Responders @19h; % (N/tot)	82 (49/60)	45 (25/56)	4 (2/56)	58 (35/60)
Δ% change @30min; median (25th;75th)	57 (37; 90)	15 (10; 43)	-9 (3; 13)	-5 (3; 9)
Δ% change @19h; median (25th;75th)	82 (47; 148)	31 (10; 59)	-	-7 (4; 12)
PaO_2:FiO_2 responders that improved Cdyn; % (N/tot)	-	37 (18/42)	-	-

Supplemental table 3

Ventilation modes used during the study.

Mode	bsl supine	30 prone	120 prone	prone before turn	30 supine,	120 supine,
PC	10	0	0	0	0	0
PRVC	2	0	0	0	0	0
PSV	6	14	13	13	13	9
SIMV	20	23	23	23	23	23
PRVC	2	0	0	0	0	0
DuoPAP	1	2	2	1	0	0
BiLevel	2	2	2	0	0	0
APRV	6	6	6	4	0	0
CPAP	2	3	3	0	0	0
iASV	0	13	13	13	13	14
MMV	0	4	4	3	0	0
VKTS	0	1	2	1	1	1
